# Irrigation levels affects biomass yields and morphometric characteristics of range grasses in arid rangelands of Kenya

**DOI:** 10.1186/s40064-016-3309-8

**Published:** 2016-09-22

**Authors:** O. K. Koech, R. N. Kinuthia, G. N. Karuku, S. M. Mureithi, R. Wanjogu

**Affiliations:** 1 Range Management Section, Department of Land Resource Management and Agricultural Technology, University of Nairobi, P.O. Box 209053-00625, Nairobi, Kenya; 2 National Irrigation Board, Mwea Irrigation Agricultural Development (MIAD) Centre, P. O. Box 210, Wanguru, 10303 Kenya

**Keywords:** Soil moisture content, Range grasses, Pasture irrigation, Semi-arid rangelands, Herbage, Biomass yields

## Abstract

**Background:**

Production of range grasses under irrigation has been widely adopted in the arid environments of Kenya as a strategy for seasonal forage supply gap. However, their productivity has only been done under conventional methods without an evaluation of their performance at varied soil moisture conditions. This information is needed for making sustainable management of irrigation water and also increased pasture productivity at the current intensification of the production systems.

**Methods:**

Aboveground biomass of six rangeland grasses (*Chloris roxburghiana*, *Eragrostis superba*, *Enteropogon macrostachyus*, *Cenchrus ciliaris*, *Chloris gayana*, and *Sorghum sudanense*) in pure and mixed stands at 80, 50 and 30 % soil moisture field capacity (FC), and control under rainfed as main plots. The main plots were divided into 30 subplots and randomly allocated ten grass species in three replicates. The moisture content was monitored by gypsum blocks which aided in irrigation times and levels. Seeds were sown by broadcast method in tractor ploughed and harrowed to fine tilt land. Biomass and growth morphometric characteristics were measured at phenological growth stages of 10, 12 and 14 weeks, representing vegetative stage, flowering and seed setting and mature with ripened seed stages for the studied range grasses.

**Results:**

All the irrigated treatment yielded significantly (p ≤ 0.05) higher above ground dry matter than the rainfed. *S. sudanense* had the highest yields at 80 % FC (13.7 t ha^−1^), though not significantly different from the 50 and 30 % FC (11.6 and 7.7 t ha^−1^), respectively. *C. gayana* and *C. roxbhurghiana* yields were not significantly affected by changes in soil moisture content with yields ranging between 10.1 and 10.8 t ha^−1^. *C. ciliaris* performed better at 50 % FC (9.1 t ha^−1^). Differences in tiller numbers across the watering treatments and grass species were not significant, but very low under rainfed conditions. The tiller heights in all the species were lower under rainfed than irrigated treatments. *S. sudanense* had the highest tiller height and biomass followed by *C. gayana* and *E. Macrostachyus*, respectively.

**Conclusion:**

Here we demonstrate that the production of range pastures under irrigation in the arid environments should consider individual species’ responses to different soil moisture content for better yields and water conservation. The results show the species of importance for consideration under irrigation systems are *S. sudanense and C. gayana.*

## Background

Rangelands are characterized by variable supply of fodder for livestock (Smith et al. 2010) which is largely attributed to low and erratic precipitation. During normal wet seasons, most of these lands support large volumes of forage which is also of relatively high quality (Mbatha and Ward 2010). The dry seasons, on the other hand, are characterized by scanty amounts forage which is mostly of poor quality (Ontitism et al. 2000). Until recently, pastoralists employed livestock mobility as the main mechanism of adapting to the feed deficits during the dry season (Orindi et al. 2007). This strategy is increasingly becoming untenable today due to a wide array of socio-economic, political and anthropogenic factors such as extension of crop farming and human settlements (cities and towns) into the Rangelands lands. These factors entail rapid land fragmentations. Consequently, in most parts, the vast tracks of Rangelands which facilitated the free movements of livestock are virtually gone. This paradigmatic shift is being compounded by the climate change phenomenon. Under these circumstances, livestock feed supply remains a major challenge and is most likely going to get worse (Kirkbride and Grahn 2008). Therefore, unless appropriate steps are urgently taken, the livelihoods of the many pastoral and agro-pastoral communities residing in these areas will continue being disrupted.

Large-scale cultivation, harvesting and storing of the forage in form of hay to be utilized during the dry season, when the open pastures have been depleted, has been cited as a potential strategy of bridging livestock feed deficits and adapting to climate change in the dry lands (USAID 2011). However, evaluation of pasture species to identify the most productive and adapted species under the arid and semi-arid Rangeland environments has not been comprehensive (Mero and Uden 1997). Interestingly, study by Craine et al. (2013) established that grasses have varied physiological tolerance to droughts and are found in broad bioclimatic ranges. Balachowski et al. (2016) also showed potential for drought tolerance among California perrenial native grasses. This presents an opportunity to exploit these grass gene resources to benefit different conditions in the variable drylands. Craine et al. (2012) also stressed on the need for research to compare the physiology of a range of grass species with under varied production constraints. Very few site-specific studies have evaluated the practical feasibility of cultivating indigenous grasses on a large scale for hay production in Kenya (Rao et al. 1996; Muhammad 1989; Mganga 2009; Mganga et al. 2010b). For this to be pursued there is need for evaluation on their productivity potential under different water regimes, to identify species with better water use efficiency (WUE) and high productivity. This is in mind of the water scarcity challenges in the dryland rangelands. Since these grasses under natural conditions grow in mixed stands, research to determine whether pure stands are more productive than mixed stands is required. If the later are more productive, then which species are most compatible? This study was therefore carried out to evaluate the Morphological growth responses (tiller number, tiller length, number of leaves per tiller and plant density) and the above ground dry matter yield (AGDM) of six indigenous range grasses, namely; *Chloris roxburghiana*, *Eragrostis superba*, *Enteropogon macrostachyus*, *Cenchrus ciliaris*, *Chloris gayana*, *Sorghum sudanense.* These grasses are the commonly found and grown in the Kenya semi arid and arid rangelands and have been promoted for production under irrigation to increase animal feed supply. This study hypothesis was that different range grass species have varied productivity under varying soil moisture levels and pure stands are more productive than mixed stands.

## Methods

### Study area

**Fig. 1:**
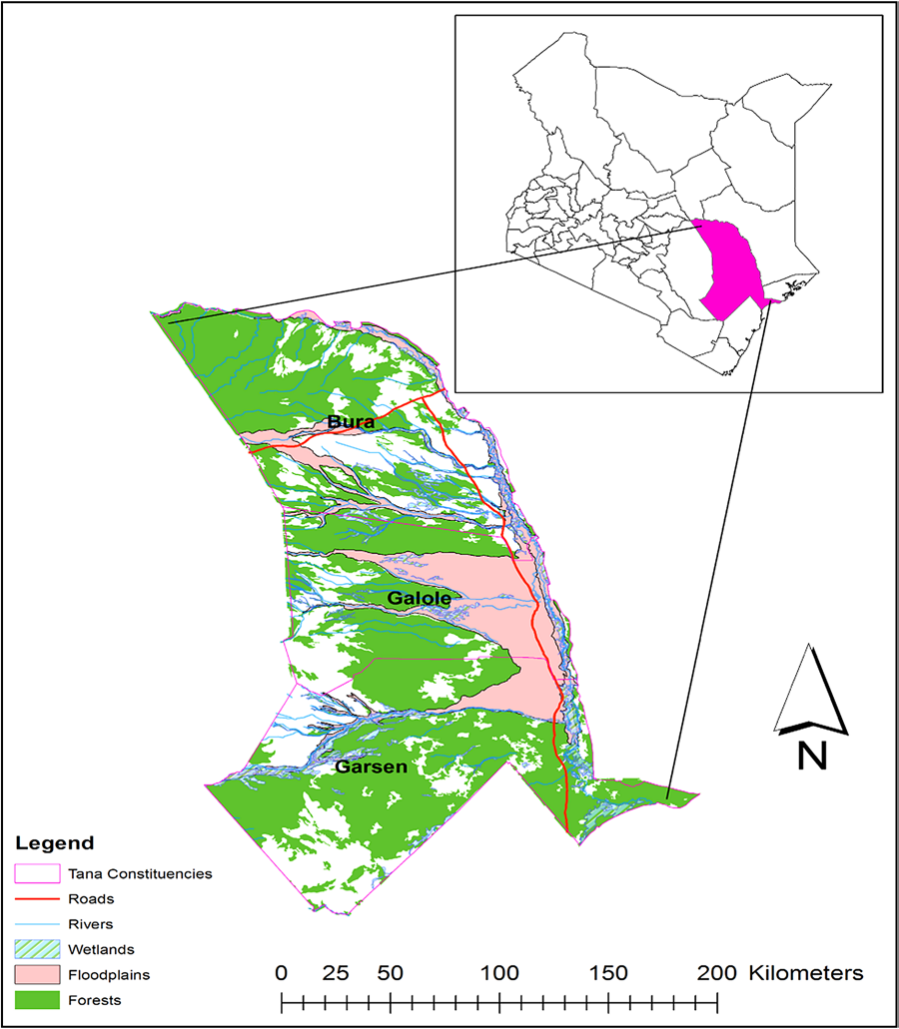
Study country—Kenya (*top right*) in relation to study area—Tana River County

The study was conducted within Bura Irrigation Scheme in Tana River County, Kenya (Fig. 1), within coordinates 1°30′S, 40°0′E, 1.5°S 40°E. The experimental period was September, 2012 to April, 2013. The study site is within the arid areas of Kenya, characterized by frequent droughts and conflicts between farmers and pastoralists over pasture and water resources. The climate is hot and dry with temperature ranges of 20–38 °C, with highest temperatures occurring during February and April and September to October. Rainfall is bimodal in distribution with long rains occurring between April–June and short rains in November–December. Long-term average rainfall range is 220–500 mm. Main soil types are vertisols and vertic fluvisols characterized by low infiltration rates, swelling and forming of ponds during the wet season (Valentin 1995; Khitrov and Rogovneva 2014). During dry seasons, the soil dry out and form wide cracks.

### Experimental design and layout

This was factorial experiment in a completely randomized design comprising two factors, grass species and soil moisture content at ten and four levels, respectively. One-acre parcel of land that had not been cultivated during the previous season was identified within the irrigation scheme. The land was cleared of all bushes, ploughed and harrowed by tractor to a fine tilth. The area was then divided into 4 main plots (39 m × 11 m) each and 5 m apart. The blocks were then randomly assigned to the watering regimes as follows: treatment one (T1) = 80 % FC, treatment two (T2) = 50 % FC, treatment three (T3) = 30 % FC and treatment four (T4) = control (rain fed). Each main plot was then sub-divided into 30 sub-plots measuring 3 m × 3 m with 1 m boundary to which the grass species treatment levels were randomly assigned as follows: *C. roxburghiana* (CR)—(T_1_), *E. superba* (ES)—(T_2_), *E. macrostachyus* (EM)—(T_3_), *C. ciliaris* (*CC*)—(T_4_), *C. gayana* (CG) (T_5_), *S. sudanense* (SB)—(T_5_), *E. superba* + *E. macrostachyus* (T_7_), *E. superba* + *E. macrostachyus* + *C. ciliaris*—(T_8_), *E. superba* + *E. macrostachyus* + *C. ciliaris* + *C. gayana*—(T_9_) and *E. superba* + *E. macrostachyus* + *C. ciliaris* + *C. gayana*; *E. superba*—(T_10_).

### Experimental materials, sowing and irrigation

**Fig. 2:**
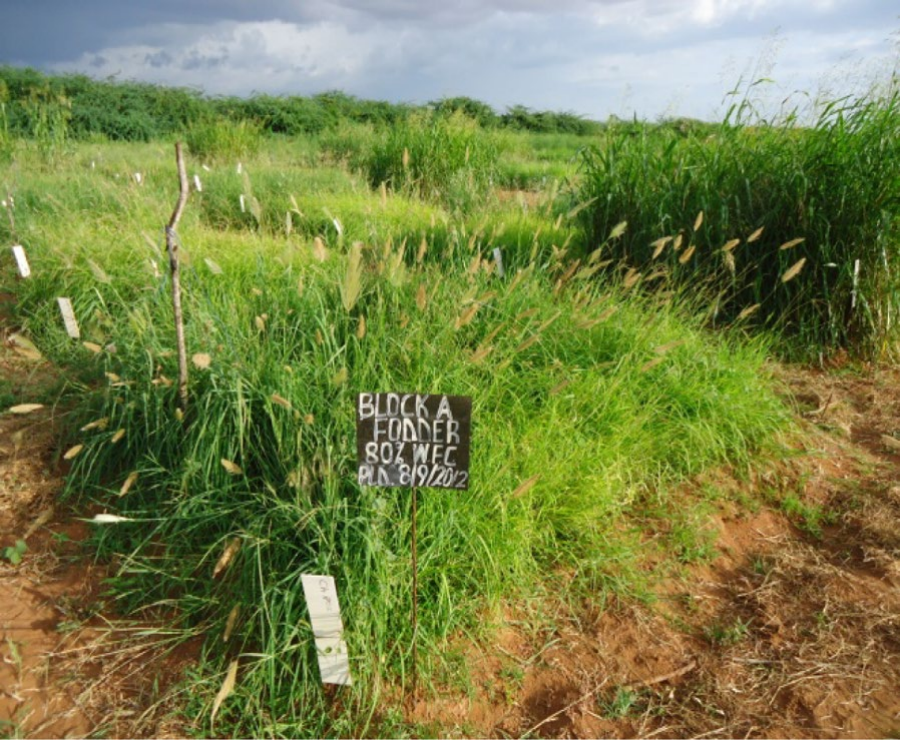
Experimental plots showing the grass species growing at their respective irrigation levels

Gypsum blocks (GBs—electrical resistance blocks) were installed at the centre of each sub plot, at two depths, 15 and 30 cm in separate holes which were dug using a 50 mm soil auger. This depth was within the root zone of the grass species under this study. Prior to installation, GBs were soaked overnight as recommended, and reading of the GBs at saturation taken. The study sites undisturbed soil samples in core rings were taken as described by Dobriyal et al. (2012) and soil moisture reading at saturation determined (100 % field capacity) (FC) readings, and the amount (mls) of moisture applied to attain this recorded. Thereafter, the amount of moisture needed to attain 30, 50 and 80 % was calculated from the amount used to attain 100 % FC. This was then used to wet the soil when GBs is placed at the centre for the calibration to the prescribed moisture level readings using the moisture meter. This was done before installation where moisture readings corresponding to 80, 50, and 30 % FC soil moisture content was calibrated for all the GBs. After installation, wire ends originating from the installed blocks were carefully supported by vertical sticks for ease of taking readings and identification of installation points. The method was also used in determining soil moisture recharge times to maintain prescribed moisture contents of 80, 50 and 30 % FC after continuous monitoring of soil moisture changes at intervals of 2–3 h from the exposed wire ends of the GBs using moisture meter. The grass seeds were sourced Kenya Agricultural and Livestock Research Organization (KALRO), Kiboko Range Research Station. The seeds were tested for germination percentage using the standard seed test by germination method as described by ISTA (1976) before planting. The determined germination rates were used to determine the mixing and sowing rates of the species. Sowing was done manually by broadcast method. Ammonium Phosphate fertilizer was applied to all the treatments at the recommended rate of 200 kg ha^−1^ to enhance establishment. Thereafter, no fertilization was done for the whole growing period. Other routine pasture husbandry practices such as weeding were done uniformly for all the treatments. Figure 2 shows the experimental plots with the grass species growing at the different soil moisture content levels.

### Data collection

#### Above ground biomass production

Above ground biomass yield was estimated at three phenological stages (12, 14, 16 weeks) representing vegetative stage, flowering and seed setting and mature with ripened seed stages for the studied range grasses (Mganga 2009; Arzani et al. 2004). The biomass measurements was done using the total harvesting method where a 0.25 m^2^ quadrat was systematically placed in each sub-plot with three replicates and all the aboveground biomass clipped to 2.5 cm stubble height each time (Tarawali et al. 1995). The harvested materials from each quadrat were stored in separate labeled brown paper bags; oven dried at 80 °C for 96 h and weighed on a digital scale (AOAC 1990). The average weights were then extrapolated to production per hectare (kg^−1^ ha^−1^).

#### Tiller heights, numbers and number of leaves per tiller

Tiller heights and numbers and species density were assessed on 12th week from sowing when the grasses were at flowering and seed setting stage. The number of tillers around three randomly selected ‘mother’ shoots were counted, the heights of each tiller measured and the number of leaves per tiller counted. Tiller heights were measured from the base to the tip of the longest leaf. These were tagged for ease of identification during subsequent assessment.

#### Species density

Plant species density was estimated by frequency grid method (Vogel and Masters 2001). Four grid sampling points were done by systematically placing the grid in each of the sub-plot and the number of each grass species inside grid identified and counted. The frequency of each species was then calculated by summing up the number of plots each species appeared from the four grid sampling points and dividing by 100 (Eq. 1). Frequencies were then multiplied by 0.4 to give a conservative estimate of density (plants m^−2^) as described by Vogel and Masters (2001) (Eq. 2). Plant density (plant m^−2^) estimates were done at 12th week from sowing.

1 $$Frequency = \frac{{ {\text{Number}}\,{\text{of}}\,{\text{quadrats}}\,{\text{in}}\,{\text{which}}\,{\text{species}}\,{\text{occur}}}}{{{\text{total}}\,{\text{number}}\,{\text{of}}\,{\text{quadrats}}\,{\text{sampled}}\,(100 )}} \times 100$$

2 $$Species\,density = {\text{Frequency}}\, ( {\text{in}}\,{\text{Eq}} .\, 1 )\times 0.4$$

### Statistical analysis

Statistical analyses included analysis of variance (ANOVA) at 95 % confidence level. Where significant differences were detected the means were separated by the least significant difference (LSD) method at 5 % probability level. Correlations analysis of AGDM yields with grass species morphometric parameters (tiller numbers, tiller heights, shoot height and number of leaves per tiller) were drawn in pure stands.

## Results and discussions

### Moisture regimes and soil moisture changes within the experiment

Table 1 presents the rainfall amount (mm), irrigation (mm), soil moisture change and evapotranspiration for the 80, 50 and 30 % FC soil moisture content and rainfed during the experimental period. There was an increase in evapotranspiration with increase in phenological growth stages among all the moisture level treatments. The cumulative rainfall amounts for the 4 months of September–December 2013 were 297.9 mm. This was the active growing period during biomass data collection and calculation AGDM yields. The low yields observed in rain fed treatments across the experimental period can be explained by the low evapotranspiration levels, due to lack of supplemental irrigation (Koech et al. 2015). The soil moisture changes were also very low within rain fed treatment compared to the irrigated plots. Morgan et al. (2001) reported seasonal moisture changes to affect leaf photosynthetic activity, with low levels negatively affecting rates of photosynthesis. These results are also supported by Fay et al. (2003), where they demonstrated that soil moisture affects carbon dioxide responses in C_4_ grassland species. The highly variable rain fall received during the experimental period echoes the reports of Knapp et al. (2002), that increased variability in precipitation patterns and soil moisture in grasslands will increase plant water stress and alter key C cycling processes such as net primary productivity. This has been shown to be a likely happening for species that were growing under rain fed conditions in this study. Altered precipitation has also been documented to affect net primary productivity in mixed grass prairies (Xu et al. 2013). Grasses have also been documented to respond to droughst during dry seasons by reducing the ability to gain carbon once water stress is attained (Craine et al. 2012).

**Table 1:** Rainfall amount (mm), irrigation (mm), soil moisture change and evapotranspiration for the 80, 50 and 30 % FC soil moisture content and rain fed treatments

Parameter	Values				
	Week 8	Week 10	Week 12	Week 14	Week 16
80 % field capacity					
Rainfall (P) (mm)	54.2	224.2	204	242.5	297.9
Irrigation (I) (mm)	367	484	556	664	770
Soil moisture change (ΔS)	147	210	214	198	144
Evapotranspiration (ET)	274.2	498.2	546	708.5	907.1
50 % field capacity					
Rainfall (P) (mm)	54.2	224.2	204	242.5	297.9
Irrigation (I) (mm)	289	366	442	524	669
Soil moisture change (ΔS)	121	187	203	113	105
Evapotranspiration (ET)	222.2	403.2	443	653.5	861.9
30 % field capacity					
Rainfall (P) (mm)	54.2	224.2	204	242.5	297.9
Irrigation (I) (mm)	211	280	310	215	456
Soil moisture change (ΔS)	98	268	225	230	265
Evapotranspiration (ET)	167.2	236.2	289	227.5	488.9
Rainfed					
Rainfall (P) (mm)	54.2	224.2	204	242.5	297.9
Irrigation (I) (mm)	0	0	0	0	0
Soil moisture change (ΔS)	33	79	85	67	113
Evapotranspiration (ET)	21.2	145.2	119	175.5	184.9

Rainfed treatment did not have supplemental irrigation and hence the zero values in the irrigation row

### Above ground dry mater (AGDM) production

The average above ground biomass yields (kg ha^−1^) of the six grass species in pure and mixed stands under 80, 50 and 30 % FC soil moisture regimes and 12, 14 and 16 weeks old is presented in Table 2. As expected, changes in the soil moisture profiles had a significant (p < 0.05) effect on the performance of all the grasses, whether in pure or mixed stands. In pure stands, at 12 weeks phonological stage within the 80 % FC soil moisture range, SB and CG species performed significantly (p < 0.05) better (>9.5 and 7.9 t ha^−1^, respectively) than all the other four species. Under 50 % FC moisture content SB and CG, EM, yielded significantly (p < 0.05) more forage (7.0–9.0 t ha^−1^) than the other three species, e.g. CR and ES with 2.5 t ha^−1^).

**Table 2:** Mean above ground biomass yields (kg ha^−1^) of the six grass species in pure and mixed stands at 80, 50 and 30 % FC at 12, 14 and 16 weeks phenological stage

	CR	ES	EM	CC	CG	SB	CR/ES	CR/ES/EM	CR/ES/EM/CC	CR/ES/EM/CC/CG
Week 12										
80 % FC	3600.4^b^ ± 76.9	3468.3^b^ ± 34.0	6600.6^b^ ± 37.7	4064.6^b^ ± 18.9	7932.2^e^ ± 93.1	9464.4^e^ ± 23.1	4000.8^b^ ± 84.1	3932.5^b^ ± 14.4	5532.7^b^ ± 11.5	9524.2^e^ ± 163.3
50 % FC	2532.3^a^ ± 68.1	1800.5^a^ ± 30.4	7400.7^e^ ± 57.7	6532.9^b^ ± 93.0	9400.6^e^ ± 44.4	9200.5^e^ ± 31.2	1464.9^a^ ± 2.9	6732.3^b^ ± 59.7	5932.1^b^ ± 66.6	7664.4^e^ ± 59.7
30 % FC	2732.3^a^ ± 17.6	3264.1^b^ ± 7.6	5400.1^b^ ± 30.4	5932.4^b^ ± 45.1	9000.7^e^ ± 50.0	7264.8^e^ ± 35.1	1200.9^a^ ± 5.0	4132.1^b^ ± 18.9	4932.3^b^ ± 7.6	6800.6^b^ ± 21.8
Rainfed	732.5^c^ ± 87.1	364.1^d^ ± 57.6	500.3^d^ ± 80.4	532.5^c^ ± 75.1	707.5^c^ ± 80.0	764.6^c^ ± 55.2	340.4^d^ ± 85.2	432.8^c^ ± 78.1	432.4^c^ ± 57.5	610.5^c^ ± 51.8
Week 14										
80 % FC	2400.2^a^ ± 13.2	4332.5^b^ ± 18.9	5132.9^b^ ± 12.6	2532.4^a^ ± 17.6	9000.3^e^ ± 47.7	12,664.7^f^ ± 25.7	8400.3^e^ ± 26.5	5264.2^b^ ± 16.1	5400.3^b^ ± 13.2	8464.4^e^ ± 44.8
50 % FC	1800.5^a^ ± 5.0	1200.6^a^ ± 5.0	9000.8^e^ ± 15.0	8400.2^e^ ± 20.0	9532.1^e^ ± 18.9	6864.2^b^ ± 128.5	1600.8^a^ ± 18.0	7864.9^e^ ± 15.3	4332.4^b^ ± 12.6	7932.3^e^ ± 81.3
30 % FC	3264.5^b^ ± 7.6	3600.6^a^ ± 5.0	6400.9^b^ ± 5.0	5332.1^b^ ± 10.4	9400.5^e^ ± 18.0	7124.3^e^ ± 10.4	1932.1^a^ ± 5.8	4064.0^b^ ± 10.4	5000.1^b^ ± 15.0	5800.7^b^ ± 26.5
Rainfed	764.2^c^ ± 57.6	381.0^d^ ± 55.1	604.3^d^ ± 45.0	558.2^c^ ± 50.4	767.0^c^ ± 48.0	824.9^c^ ± 60.4	532.8^c^ ± 55.5	464.8^c^ ± 60.3	511.0^c^ ± 65.1	680.6^c^ ± 56.5
Week 16										
80 % FC	3320.6^b^ ± 9.8	5600.3^b^ ± 13.2	6464.5^b^ ± 20.8	2464.8^a^ ± 24.7	10,864.1^e^ ± 41.9	13,664.2^f^ ± 17.6	8864.8^e^ ± 34.03	5600.9^b^ ± 13.2	5732.5^b^ ± 15.3	9664.2^e^ ± 106.1
50 % FC	2532.3^a^ ± 10.4	1532.2^a^ ± 12.5	10,464.4^e^ ± 17.6	9132.6^e^ ± 34.0	10,200.1^e^ ± 25.0	11,600.0^e^ ± 17.3	1800.5^a^ ± 5.0	7332.1^e^ ± 59.2	6200.8^b^ ± 37.8	9264.6^e^ ± 20.0
30 % FC	2132.6^a^ ± 12.6	3132.7^b^ ± 12.6	6664.8^b^ ± 10.4	6864.8^b^ ± 12.6	10,132.1^e^ ± 7.6	7664.5^e^ ± 10.4	2864.3^a^ ± 12.6	4400.2^b^ ± 18.0	5132.1^b^ ± 2.9	6800.0b ± 15.0
Rainfed	759.8^c^ ± 62.2	372.3^d^ ± 42.6	664.8^c^ ± 50.4	664.5^c^ ± 42.5	832.7^c^ ± 3 7.6	964.8^c^ ± 60.5	764.5^c^ ± 42.5	480.1^c^ ± 48.0	532.4^c^ ± 32.2	702.3^c^ ± 45.1

Means within the same columns with different superscripts are significantly different at p < 0.05

*CR C. roxburghiana*, *ES E. superba*, *EM E. macrostachyus*, *CC C. ciliaris*, *CG C. gayana*, *SB S. sudanense*, *FC field capacity*, ± *standard deviation*

At the 30 % FC moisture profile, CG was the most prolific (>9.0 t ha^−1^) followed by SB (7.2 t ha^−1^). At the 14 weeks and 80 % FC, CG and SB were still the most prolific (>9.0 and 12.0 t ha^−1^) compared to CC and CR which yielded slightly about 2.0 t ha^−1^. Around 50 % FC moisture, EM’s performance dropped significantly to 6.7 t ha^−1^ compared to CC and CG (>8.0 and 9.0 t ha^−1^). Within 30 % FC moisture regime, CG and SB species were the most prolific (9.4 and 7.1 t ha^−1^). At about 16 weeks old (maturity) and 80 % FC, SB and CG exhibited higher performance (>10.0 and 13.0 t ha^−1^, respectively) than the rest. CC was the poorest yielding about 2.5 t ha^−1^. Within 50 % FC moisture level, EM, CC, CG and SB out-performed the other two (9.0–12.0 vs 1.5–2.5 t ha^−1^). Under 30 % FC moisture treatment, CG performed much better than all the other species (>10.0 vs <7.0 t ha^−1^). Under the rainfed conditions CR, CG and SB were consistently the most productive species although they produced <10 % what any of the irrigation treatments produced. For the mixed species treatments, the five-species treatment combination performed consistently higher (p ≤ 0.05) than the two-, three- or four- species combinations. However, there were unexplained variations among the two and three mixed species in terms of yields declining or increasing at higher soil moisture levels. This could have been a result of complex inter-specific completion responses, which was not keenly studied in this study, and may need to be further evaluated with this respect. Wetzel and Van Der Valk (1998) also reported plant response to environmental factors, intra-specific competition, nutrient levels and inter-specific competition to be important factors affecting productivity. But notably also, the study reveals some species like *C. ciliaris* and *C. gayana* were performing poorer under high (80 % FC) moisture content.

The high biomass yields of *E. macrostachyus* and *S. sudanense* could be attributed to the faster germination rates observed in this study that gave them a head start in accumulating biomass at early growth stages (Koech et al. 2014). The faster germination rate of seeds allows the plant to have a competitive advantage over the slow germinating plants due to the enhanced competitive advantage for resources (Kadmon and Schimida 1990). The differences in biomass production for the species at the different growth stages could be attributed to the morphological differences of the grasses. *S. sudanense* tent to be stemmier and was able to maintain higher vegetative productivity, which accounts for the higher AGDM yields recorded at all the three soil moisture content. The same species had higher tiller numbers and tiller height compared to the other species which also explains the outperformance of the species amongst the rest. *S. sudanense* had deeper roots than all the species and this may have allowed the plant to utilize deeper soil moisture even under lower soil moisture content as reported in the same study under a different objective (Koech et al. 2014). *S. sudanense* has also been observed to have higher WUE, higher production capacity per unit area and tolerance to drought and high temperatures (Uzun et al. 2009).

The higher AGDM yields of >10 t ha^−1^ observed in *S. sudanense* at all the three soil moisture content concur with those of Ferat et al. (2009), who reported the lowest yields for *S. sudanense* variety named Chopper to be 11.68 t ha^−1^ under irrigation.

The lower AGDM production exhibited by *C. ciliaris* at 80 % FC can be attributed to the species low tolerance for high soil moisture and water logging conditions (Jacobs et al. 2004). This was observed from the slow percolation of water at this moisture level which took 30–45 min to clear from the soil surface after irrigation. The slow infiltration in the area may be a result of a hard pan below the soil profile, even though this was not tested, but the area was previously cultivated using tractors and irrigated which are known to cause sub-surface hardpans. However, other factors like the high clay content in the study are vertisols could also have contributed to the low infiltration (Yaalon and Kalmar 1978; Waiser et al. 2007). The phenomenal increase in AGDM for *C. ciliaris* at lower soil moisture content can be attributed to the species drought tolerance and hence the resultant higher AGDM (Heuzé et al. 2013). Akram et al. (2008) identified *C. ciliaris* as drought tolerant and having ability to accumulate N, P, K^+^ and Ca^2+^ during growth which enhanced its productivity at lower moisture levels. The species has also been reported to produce higher biomass (14.6 t ha^−1^) under irrigation and high WUE (0.7 kg ha^−1^ m^−3^) in the United Arab Emirates in sandy loam soils which was similar to this study (Osman et al. 2008). However, the yields were higher than what was realized in the present study, which could be due to the difference irrigation methods used.

The higher yields of *C. gayana* at the three soil moisture contents compared to *C. ciliaris* contradict the observations of Asadullah and Ahmed (2010), who reported the latter to perform better than the former in water utilization. However, their study focused on moisture extraction capacities and not the biomass yields. All the same, *C. gayana* have performed better than *C. ciliaris* maybe due to the higher germination rates which might have given it a competitive advantage (Koech et al. 2014). The ability of the species to germinate faster allows for quicker and faster penetration of roots to deeper soil profiles and thus increasing the volume of soil from which water is extracted and hence drought tolerance (Bibi et al. 2010). Similar results of higher yield in *C. gayana* than *C. ciliaris* and *E. superba* with fast germination were reported by Bulle et al. (2010) in study done Northern Kenya under rainfed conditions. Ontitism et al. (2000) also reported potential of *C. gayana* to be higher for livestock feed in dry rangelands. Likewise, the higher AGDM biomass yields depicted by *C. gayana* of over 10 t ha^−1^across all the three soil moisture content can be attributed to its adaptability to both low and high soil moisture content similarly to *S. sudanense*. This gives them an advantage over many range grass species in the semi-arid rangelands and hence greater potential for pasture establishment and reseeding denuded rangelands.

The extremely low productivity of all the six grass species under rainfed treatment (control) was attributed to the low and poor distribution of precipitation received in the study area during the 4 months growing season (297.9 mm with 149.8 mm received within 3 days) (Table 1). The slightly higher yields shown by *S. sudanense* under rainfed over the other five species is attributed to its faster germination rate, deep rooting which averaged about 43.7 cm compared to the other species that ranged between 16 and 38 cm. This trait may have enabled *S. sudanense* to utilize water on the deeper subsoil profile which is known to dry at a slower rate during the dry seasons. Snyman and Joubert (1996), also reported *S. Sudanense* to have deeper rooting system that enhances water absorption.

The high AGDM in the five grass species mixtures than monocultures can be attributed to the functional diversity effect (Berdahl et al. 2001; Thompson 2013). This is basically the contribution of each species to the total biomass yield based on their growth characteristic and morphological traits which play a major role in determining the total yields from the varied adaptability capacities. These findings concur with the study by Mganga et al. (2010a) working with *C. ciliaris*, *E. superba*, and *E. machrostachyus* grown in pure stands and in mixtures. They reported mixture stands to have higher yields compared to pure stands and attributed this to growth characteristics and morphological traits of the individual species under mixtures. Similar observations were also reported by Thompson (2013), working with grass-alfalfa mixtures and orchardgrass and fescue mixtures and observed mixtures to have outperformed alfalfa, orchardgrass, and tall fescue monocultures by 12 %, which was related to functional diversity effects. The fast growing species like *E. macrostachyus* and *C. gayana* contributes to faster buildup of biomass at the early growing stages in mixtures. When grass species are grown in mixtures in the natural environment, some species dominate others due to competitive abilities for water and nutrients but that is not certain since climatic and environmental conditions always come into play (Bergh 1968). This study observation was done in a short period and we cannot confidently conclude that five species mixture level has higher yields and therefore there is need for further studies to observe the responses over longer periods. Ideally, it was expected that mixed stands would have more competition and hence lower yields, but the contrary was observed. There is need for further research on what really happens under mixtures in influencing the grass biomass yields.

### Effect of soil moisture on the morphometric parameters of the grasses

The tiller numbers and lengths; number of leaves per tiller and plant density by grass species and soil moisture content levels (80, 50, 30 % FC and rain-fed) are presented in Table 3. Tiller numbers and tiller lengths of a specific grass species did not have significant difference at the different soil moisture. However, there was a significantly lower (p ≤ 0.05) tiller numbers in rainfed treatment compared to the three soil moisture levels for all the grasses. The tiller lengths and shoot height showed increasing trend for all the species which was expected since they develop and increase in length with plant maturity. SB had the highest number of tillers at all the three soil moisture content. The number of leaves per tiller for specific grass species did not significantly vary across all the soil moisture content and the rainfed treatment. There was significant difference (p ≤ 0.05) in plant density between the six grass species; however, this was not significantly different for the specific grass species at the different soil moisture content. SB had the highest plant density followed by CG and EM.

**Table 3:** Mean tiller number, tiller length (cm), shoot length, plant density and leaves per tiller of six grass species at 80, 50 and 30 % FC soil moisture content at week 12 phenological stage

	CR	ES	EM	CC	CG	SB
Tiller numbers						
80 % FC	11.1^b^ ± 2.3	19.3^b^ ± 4.7	16.2^b^ ± 2.3	28.0^b^ ± 6.4	21.1^b^ ± 11.1	31.0^b^ ± 16.1
50 % FC	10.8^b^ ± 3.3	14.3^b^ ± 5.8	14.1^ab^ ± 3.3	22.0^ab^ ± 11.4	18.1^ab^ ± 9.1	29.0^b^ ± 6.1
30 % FC	12.3^ab^ ± 6.3	18.3^b^ ± 7.1	12.1^ab^ ± 2.4	20.0^ab^ ± 8.4	16.3^ab^ ± 9.4	31.2^b^ ± 11.1
Rainfed	3.3^a^ ± 1.3	3.1^a^ ± 1.1	4.1^a^ ± 2.3	4.7^a^ ± 2.4	4.3^a^ ± 1.2	5.2^a^ ± 2.1
Tiller length						
80 % FC	98.9^c^ ± 23.1	111.9^c^ ± 54.8	89.7^c^ ± 13.8	76.8^ab^ ± 12.7	114.8^b^ ± 21.9	119.6^b^ ± 67.9
50 % FC	57.9^b^ ± 21.4	99.7^b^ ± 47.3	74.3^b^ ± 32.5	96.5^b^ ± 44.2	102.8^b^ ± 54.3	128.6^b^ ± 57.1
30 % FC	64.6^b^ ± 31.3	97.9^b^ ± 41.1	77.7^b^ ± 33.3	100.8^e^ ± 56.8	109.8^b^ ± 51.3	143.3^c^ ± 54.7
Rainfed	24.6^a^ ± 11.2	33.1^a^ ± 21.6	41.3^a^ ± 11.3	33.2^a^ ± 18.1	43.3^a^ ± 19.5	52.4^a^ ± 24.3
Leaves/tiller						
80 % FC	4.4^a^ ± 2.1	6.9^a^ ± 2.1	5.1^a^ ± 2.1	7.8^b^ ± 2.8	8.1^a^ ± 3.1	6.9^a^ ± 2.2
50 % FC	4.4^a^ ± 2.1	6.9^a^ ± 2.1	5.2^a^ ± 2.1	8.6^b^ ± 2.8	8.5^a^ ± 2.1	4.8^a^ ± 2.1
30 % FC	3.4^a^ ± 1.1	7.1^a^ ± 4.1	6.2^a^ ± 3.3	9.2^b^ ± 3.8	8.1^a^ ± 3.2	6.1^a^ ± 2.2
Rainfed	3.5^a^ ± 2.1	4.1^a^ ± 2.1	4.2^a^ ± 2.2	4.6^a^ ± 2.8	5.2^a^ ± 2.2	5.1^a^ ± 2.5
Plant density (plant m^−2^)						
80 % FC	21.2^b^	24.4^ab^	32.4^b^	27.6^a^	27.6^b^	32.8^b^
50 % FC	20.8^b^	20.4^ab^	31.6^b^	25.2^a^	28.4^b^	34.8^b^
30 % FC	22.0^b^	26.8^ab^	29.2^ab^	26.0^a^	28.8^b^	30.0^b^
Rainfed	18.0^a^	19.0a	17.2^a^	22.0^a^	20.9^a^	23.3^a^

Means within the same columns with different superscripts are significantly different at p < 0.05

*CR C. roxburghiana*, *ES E. superba*, *EM E. macrostachyus*, *CC C. ciliaris*, *CG C. gayana*, *SB S. sudanense*, *FC field capacity*, ± *standard deviation*

The observed differences in tiller numbers and heights can be attributed to the morphological growth differences associated with genetic constitution of the grass species. The outperformance of *S. sudanense* in terms heights could be attributed to the adaptability of the species by faster germination and adaptation to dry conditions (Grismer 2001).

The reduction in height in all the grass species under rainfed conditions was also observed by Jeremiah et al. (2013), working with switch grass and miscanthus species under irrigation and rainfed conditions in California, USA. Their finding on the variation responses of individual species under rainfed conditions and irrigation concurs with the findings in this study where a reduction in shoot growth was noted. They observed that switchgrass under rainfed to have outperformed miscanthus both shoot and root growth. These findings explain the variations in shoot heights amongst the six species under rainfed condition. *S. sudanense* and *E. macrostachyus* have vertical growth habits with strong and thicker culms and hence have advantage over the semi-erect species like *C. gayana* and *E. superba.* Grass heights have been shown to give grasses as a competitive advantage in resource utilization and survival. Kanak et al. (2013) reported heights to enhance grass performance in mixtures by the ability to shade other plants and reduce competition for resources. This might have been the case under the five species mixture where *C. gayana* and *E. macrostachyus* that had greater height was present and contributed more to the total AGDM yields.

The observed increase in tiller lengths and shoot height for all the grass species was expected since the tillers develop and increase in length with plant maturity just like the shoot growth. Contradicting results were observed by Ogillo et al. (2010), who reported no significant difference in tillers among range grass species at all growth stages, this may be attributed to the differences in soil types and soil moisture levels in the two study sites. Site conditions, soil type and rainfall amounts have also been reported to determine the success of establishment and hence growth responses (Opiyo 2007). Tiller numbers are important for grass plants adaptability and survival under grazing pressure since they determine photosynthetic rates and ultimately the food reserves (Laidlaw 2005). The number of tillers and leaves in a grass plant determines the rate of biomass accumulation and the quality of forage (Skinner and Moore 2007). Tillers contain leaves which are easily digestible and more preferred due to less structural carbohydrates, especially the newly developed tillers with young leaves (Wilson et al. 1991). However, a tiller will have both old leaves from previous growth season and young leaves from current season. The old leaves are lower in quality but contribute to the total biomass yields (Soininen et al. 2010), while the young ones improve on the quality. The lower tiller numbers under rainfed treatment can be attributed to acute water deficit during the growing season and hence leaf firing. On the other hand, the increase in tiller numbers for irrigated treatments can be attributed to adequate water supply that enhanced tiller recruitment. These findings are contrary to those of Teague and Dowhower (2002) working on *Bothriochloa ischaemum* under rainfed and rainfed plus 25 mm/week of supplementary irrigation. They reported soil moisture content had no effects on tiller numbers. This observation could have been from the variation in climatic conditions since they were working in a humid climate unlike the semi-arid environment in the present study. The differences in the genetic could also be the reason. However, the findings of Mganga et al. (2010b) concurred with the results of this study where tiller numbers varied with species and growth stages. Water plays an important role in nutrient absorption and translocation by plants as well as maintaining plant temperature through transpiration, and this could have been a challenge under rainfed condition hence affecting the grasses leaf and tiller development.

Genetic differences contribute to differences in tiller and leaf numbers and this could probably be the reason in this study where grass species were not exposed to other factors that are known to influence tillering such as grazing responses by grasses as an adaptation strategy. Sarukhan et al. (1984) pointed out that grass species with higher number of tillers make greater contribution to the next generation of grass species in the sward. *S. sudanense*, *C. ciliaris* and *C. gayana* had higher advantage amongst the six species under the three soil moisture content in terms of tiller production and this could be attributed to their genetic constitution and the favourable environment under irrigation. Other studies have also reported tillers to increase chances of plant survival and productivity (Skerman and Riveros 1990; Laidlaw 2005). Opiyo et al. (2011) working with *E. superba*, also observed an increase in leaf numbers with growth. Leaf dynamics in grasses are a function of new leaf development and death of leaves which are determined by the environment and genetics of the grasses (Harper 1989; Huibert and Jan 1998). However, there is a close relationship between tiller numbers and leaf recruitment per tiller (Matthew et al. 2000). Plant leaves determine the quality of forage for livestock with higher young and green leaves contributing much to the increase in crude protein (Michel and Helene 2000; Arzani et al. 2001). The leaf:stem ratio becomes a parameter of concern when analyzing for pasture quality, hence grass species with higher proportion of leaves per tiller and less stems tend to be of better quality (Ball et al. 2001; Rad et al. 2013). Therefore the higher leaf numbers in *C. gayana* and *C. ciliaris* suggest better quality forage grasses.

## Conclusion

The findings indicate the importance of soil moisture in determining grass species AGDM yields. The present reliance on rainfall in the semi-arid rangelands of Kenya for pasture productivity is the reason behind the low productivity of both pastures and livestock. Species like *S. sudanense*, *C. gayana* and *E. macrostachyus* produce over 10 t ha^−1^ which is an equivalent of about 700 bales of hay each is weighing 14 kgs under lower soil moisture content. Also, it is worth noting that species such as *C. ciliaris* are affected by higher irrigation levels (80 % FC) and lower levels (30 %), but performs better under medium (50 % FC), therefore production of range pastures under irrigation should consider individual species responses to different soil moisture content. *S. sudanense* has high biomass productivity both under high and low soil moisture content while *C. gayana* biomass productivity is not compromised by moisture levels compared to the other four grass species. The two species are the better choice for pasture production under irrigation in moisture deficit environments.

## References

[CR1] Akram NA, Shahbaz M, Ashraf M (2008). Nutrient acquisition in differentially adapted populations of *Cynondon dactylon* (L.) Per. and *Cenchrus ciliaris* L. under drought stress. Pak J Bot.

[CR2] AOAC (1990). Official methods of analysis.

[CR3] Arzani H, Torkan J, Jafari M, Nikkhah A (2001). Investigation on effects of phenological stages and environmental factors (soil and climate) on forage quality of some important range species. J Agric Sci.

[CR4] Arzani H, Zohdi M, Fish E, Zahedi Amiri GH, Nikkhah A, Wester D (2004). Phenological effects on forage quality of five grass species. J Range Manag.

[CR5] Asadullah ALA, Ahmed AS (2010). Soil water uptake efficiency of some irrigated indigenous and exotic forage species under desert climatic conditions. Asian J Sci Technol.

[CR6] Balachowski JA, Bristiel PM, Volaire FA (2016). Summer dormancy, drought survival and functional resource acquisition strategies in California perennial grasses. Ann Bot.

[CR7] Ball DM, Collins M, Lacefield GD, Maitin NP, Mertens DA, Olson KE, Wolf MW (2001) Understanding forage quality. American Farm Bureau Federation, Publication 1-01, Park Ridge

[CR8] Berdahl JD, Karn JF, Hendrickson JR (2001). Dry matter yields of cool-season grass monocultures and grass–alfalfa binary mixtures. Agron J.

[CR9] Bergh JP (1968). An analysis of yields of grasses in mixed and pure stands. Versl Landbouwk Onderz.

[CR10] Bibi A, Sadaqat HA, Akram HM (2010). Physiological and agronomic response of *Sorghum sudanense* to water stress. J Agric Res.

[CR11] Bulle H, Mamo M, Geikuku P (2010). Comparative evaluation of *Chrolis gayana* (Kunth), *Eragrostis superba* (Peyr) and *Cenchrus ciliaris* (L.) for pasture production in Marsabit central district, northern Kenya. In: Proceedings of the 12th KARI biennial scientific conference, 8th–12th November, Nairobi, Kenya, pp 826–830

[CR12] Craine JM, Nipper JB, Elmore AJ, Skibbe AM, Hutchinson SL, Brunsell NA (2012). Timing of climate variability and grassland productivity. Proc Natl Acad Sci.

[CR13] Craine JM, Ocheltree TW, Nippert JB, Town EG, Skibb AM, Kembel SW, Fargione JE (2013). Global diversity of drought tolerance and grassland climate-change resilience. Nat Clim Chang.

[CR14] Dobriyal P, Qureshi A, Badola R, Hussain SA (2012). A review of the methods available for estimating soil moisture and its implications for water resource management. J Hydrol.

[CR15] Fay PA, Carlisle JD, Knapp AK, Blair JM, Collins SL (2003). Productivity responses to altered rainfall patterns in a C4-dominated grassland. Oecologia.

[CR16] Ferat U, Serdal U, Mehmet S (2009). Yield, nutritional and chemical properties of some sorghum × *Sorghum sudanense* hybrids [*Sorghum bicolor* (L.) *Moench* × *Sorghum sudanense* Stapf.]. J Anim Vet Adv.

[CR17] Grismer M (2001). Sudangrass uses water at rates similar to alfalfa, depending on location. Calif Agric.

[CR18] Harper JL, Russell G, Marshall B, Jarvis DG (1989). Canopies as populations. Plant canopies: their growth form and function.

[CR19] Heuzé V, Tran G, Baumont R (2013) Buffel grass (*Cenchrus ciliaris*). Feedipedia.org. a programme by INRA, CIRAD, AFZ and FAO. http://www.feedipedia.org/node/482. Last updated on 23 Aug 2013, Accessed 12 Dec 2013

[CR20] Huibert JB, Jan HN (1998) Morphological analysis of leaf and tiller number dynamics of wheat (*Triticum aestivum* L.): responses to temperature and light intensity. Ann Bot 81:131–139. http://aob.oxfordjournals.org/content/81/1/131.full.pdf. Accessed 9 Apr 2013

[CR21] ISTA (1976). International rules for seed testing. Seed Sci Technol.

[CR22] Jacobs SS, van Niekerk WA, Coertze RJ (2004). Qualitative evaluation of *Cenchrus ciliaris* cv. Molopo and Gayndah as forage. S Afr J Anim Sci.

[CR23] Jeremiah JM, Jacob NB, Guy BK (2013). Root system dynamics of *Miscanthus* × *giganteus* and *Panicum virgatum* in response to rainfed and irrigated conditions in California. Bio Energy Res.

[CR24] Kadmon R, Schimida A (1990). Patterns of spatial variation in the reproductive success of a desert annual. Oecologia.

[CR25] Kanak AR, Khan MJ, Debi MR, Khandakar ZH, Pikar MK (2013). Comparison on biomass production of three fodder germplasms. Bangladesh J Anim Sci.

[CR26] Khitrov NB, Rogovneva LV (2014). Vertisols and vertic soils of the middle and lower Volga regions. Eurasian Soil Sci.

[CR27] Kirkbride M, Grahn R (2008). Survival of the fittest: pastoralism and climate change in East Africa. Oxfam Policy Pract Agric Food Land.

[CR28] Knapp AK, Fay PA, Blair JM, Collins SL, Smith MD, Carlisle JD, McCarron JK (2002). Rainfall variability, carbon cycling, and plant species diversity in a mesic grassland. Science.

[CR29] Koech OK, Kinuthia RN, Mureithi SM, Karuku G, Wanjogu R (2014). Effect of different soil water content and seed storage on quality of six range grasses in the semi-arid ecosystems of Kenya. Environ Ecol Res.

[CR30] Koech OK, Kinuthia RN, Karuku GN, Mureithi SM, Wanjogu R (2015). Water use efficiency of six rangeland grasses under varied soil moisture content levels in the arid Tana River County, Kenya. Afr J Environ Sci Technol.

[CR31] Laidlaw AS (2005). The relationship between tiller appearance in spring and contribution of dry matter yield in perennial ryegrass (*Lolium perenne* L.) cultivars differing in heading date. Grass Forage Sci.

[CR32] Matthew C, Assuero SG, Black CK, Lemaire G, Hodgson J, de Moraes A, Carvalho PCF, Nabinger C (2000). Tiller dynamics of grazed 7 swards. Grassland ecophysiology and grazing ecology.

[CR33] Mbatha KR, Ward D (2010). The effects of grazing, fire, nitrogen and water availability on nutritional quality of grass in semi-arid savanna, South Africa. J Arid Environ.

[CR34] Mero RN, Uden P (1997). Promising tropical grasses and legumes as feed resources in central Tanzania I. Effect of different cutting patterns on production and nutritive value of six grasses and six legumes. Trop Grassl.

[CR35] Mganga KZ (2009) Impact of grass reseeding technology on rehabilitation of the degraded rangelands: a case study of Kibwezi district, Kenya. M.Sc. thesis, University of Nairobi, Nairobi, Kenya

[CR36] Mganga KZ, Musimba NK, Nyangito MM (2010). The role of moisture in the successful rehabilitation of denuded patches of a semi-arid environment in Kenya. J Environ Sci Technol.

[CR37] Mganga KZ, Musimba NK, Nyariki DM (2010). Dry matter yields and hydrological properties of three perennial grasses of a semi-arid environment in east Africa. Afr J Plant Sci.

[CR38] Michel D, Helene D (2000). Growth and senescence of the successive grass leaves on a tiller. Ontogenic development and effect of temperature. Ann Bot.

[CR39] Morgan JA, Lecain DR, Mosier AR, Milchunas DG (2001). Elevated CO2 enhances water relations and productivity and affects gas exchange in C3 and C4 grasses of the Colorado shortgrass steppe. Glob Chang Biol.

[CR40] Muhammad N (1989) Rangeland management in Pakistan. ICIMOD senior fellowship series No. 1. Pan Graphics (Pvt.) Ltd., Islamabad

[CR41] Ogillo BP, Nyangito MM, Nyariki DM (2010) A comparison of two micro-catchment technologies on above ground biomass production and financial returns of three range grasses in southern Kenya, pp 849–854. http://tinyurl.com/orrvglq. Accessed 15th May 2012

[CR42] Ontitism SM, Ondabu N, Ouda J (2000) The performance and composition of ley grasses and legumes, sweet potatoes and fodder trees. In: Wamae L, Murithi F, Wasike W (eds) Proceedings of the 7th KARI biennial scientific conference held on 13th–17th November 2000, Nairobi, pp 389–392

[CR43] Opiyo OFE (2007) Land treatment effects on morphometric characters of Three grass species and economic returns from reseeding in Kitui district, Kenya. MSc. thesis, University of Nairobi

[CR44] Opiyo FEO, Ekaya WN, Nyariki DM (2011). Seedbed preparation influence on morphomeric characteristics of perennial grasses of a semi-arid rangeland in Kenya. Afr J Plant Sci.

[CR45] Orindi VA, Nyong A, Herrero M (2007) Pastoral livelihood adaptation to drought and institutional interventions in Kenya. Human Development Report Office, Occasional Paper, 54. http://www.ilri.org/Link/Publications/orindi_nyong_herrero.pdf

[CR46] Osman AE, Makawi M, Ahmed R (2008). Potential of the indigenous desert grasses of the Arabian Peninsula for forage production in a water-scarce region. Grass Forage Sci.

[CR47] Rad MS, Rad JS, da Silva JAT (2013). Forage quality of two halophytic species, *Aeluropus lagopoides* and *Aeluropus littoralis*, in two phenological stages. Int J Agron Plant Prod.

[CR48] Rao AS, Singh KC, White GR (1996). Productivity of *Cenchrus ciliaris* in relation to rainfall and fertilization. J Range Manag.

[CR49] Sarukhan J, Martinez-Ramos M, Pinero D, Sarukhan J, Dirzo R (1984). The analysis of demographic variability at the individual level and its population consequences. Perspectives on population ecology.

[CR50] Skerman PJ, Riveros F (1990). Tropical grasses.

[CR51] Skinner HR, Moore KJ, Barnes RF, Nelson CJ, Moore KJ, Collins M (2007). Growth and development of forage plant. Forages: the science of grassland agriculture.

[CR52] Smith P, Gregory PJ, Van Vuuren D (2010). Competition for land. Philos Trans R Soc B Biol Sci.

[CR53] Snyman LD, Joubert HW (1996). Effect of maturity stage and method of preservation on the yield and quality of forage sorghum. Anim Feed Sci Technol.

[CR54] Soininen EM, Hübner CE, Jónsdóttir IS (2010). Food selection by barnacle geese (*Branta leucopsis*) in an Arctic pre-breeding area. Polar Res.

[CR55] Tarawali SA, Tarawali G, Larbi A (1995) Methods for the evaluation of legumes, grasses and fodder trees for use as livestock feed. ILRI manual 1. International Livestock Research Institute (ILRI), Nairobi, p 51

[CR56] Teague WR, Dowhower SL (2002). Irrigation impact on harvest efficiency in grazed Old World Bluestem. J Range Manag.

[CR57] Thompson D (2013). Yield and nutritive value of irrigated tall fescue compared with orchardgrass: in monocultures or mixed with alfalfa. Can J Plant Sci.

[CR58] USAID (2011) Drylands livestock development program. http://kenya.usaid.gov/programs/economic-growth/412. Accessed 26 Sept 2011

[CR59] Uzun F, Ugur S, Sulak M (2009). Yield, nutritional and chemical properties of some sorghum × Sudan grass hybrids [*Sorghum bicolour* (L.) Moench × *Sorghum sudanense* Stapf.]. J Anim Vet Adv.

[CR60] Valentin C, So H, Smith GD, Raine SR, Schafer BM, Loch RJ (1995). Sealing, crusting and hardsetting soils in Sahelian agriculture. Sealing, crusting and hardsetting soils: productivity and conservation.

[CR61] Vogel KP, Masters RA (2001). Frequency grid—a simple tool for measuring grassland establishment. J Range Manag.

[CR62] Waiser TH, Morgan CL, Brown DJ, Hallmark CT (2007). In situ characterization of soil clay content with visible near-infrared diffuse reflectance spectroscopy. Soil Sci Soc Am J.

[CR63] Wetzel PR, Van Der Valk AG (1998). Effects of nutrient and soil moisture on competition between shape *Carex stricta*, shape *Phalaris arundinacea*, and shape *Typha latifolia*. Plant Ecol.

[CR64] Wilson JR, Deinum B, Engels FM (1991). Temperature effects on anatomy and digestibility of leaf and stem of tropical and temperate forage species. Neth J Agric Sci.

[CR65] Xu X, Sherry RA, Niu S, Li D, Luo Y (2013). Net primary productivity and rain-use efficiency as affected by warming, altered precipitation, and clipping in a mixed grass prairie. Glob Chang Biol.

[CR66] Yaalon DH, Kalmar D (1978). Dynamics of cracking and swelling clay soils: displacement of skeletal grains, optimum depth of slickensides, and rate of intrapedonic turbation. Earth Surf Process.

